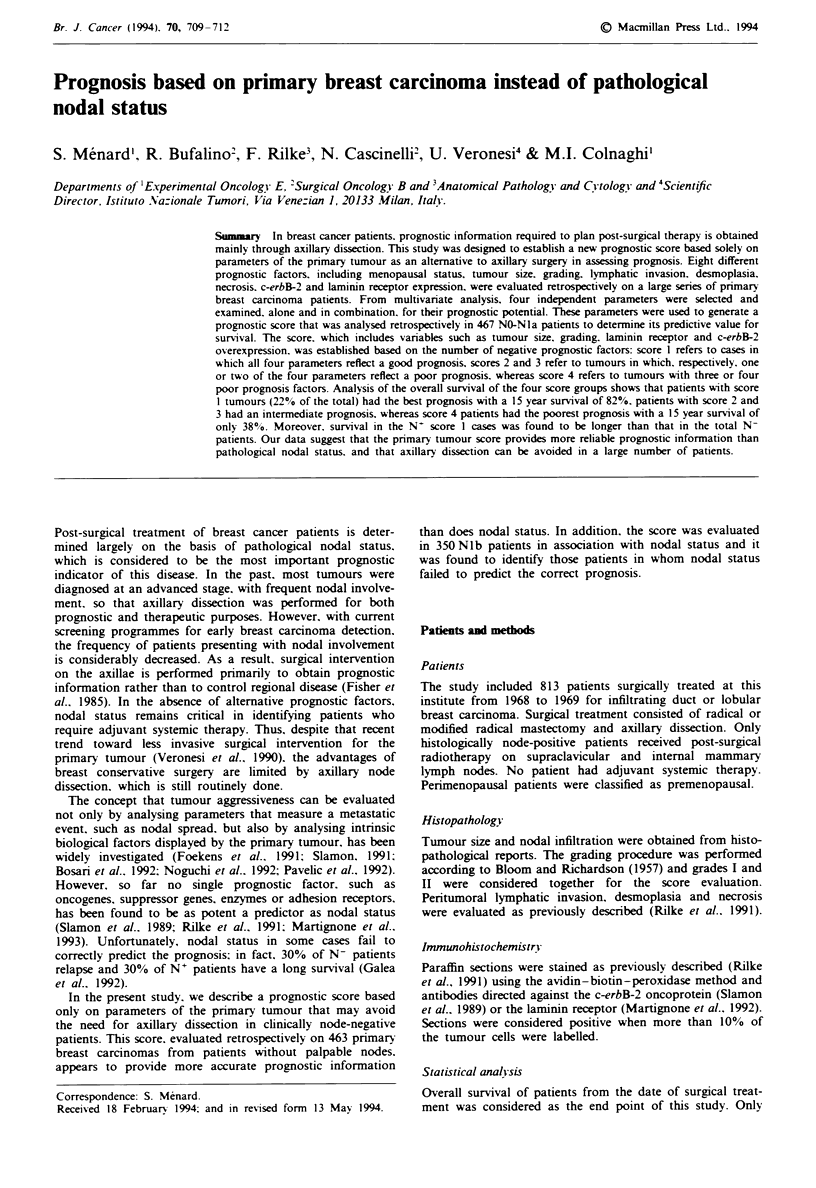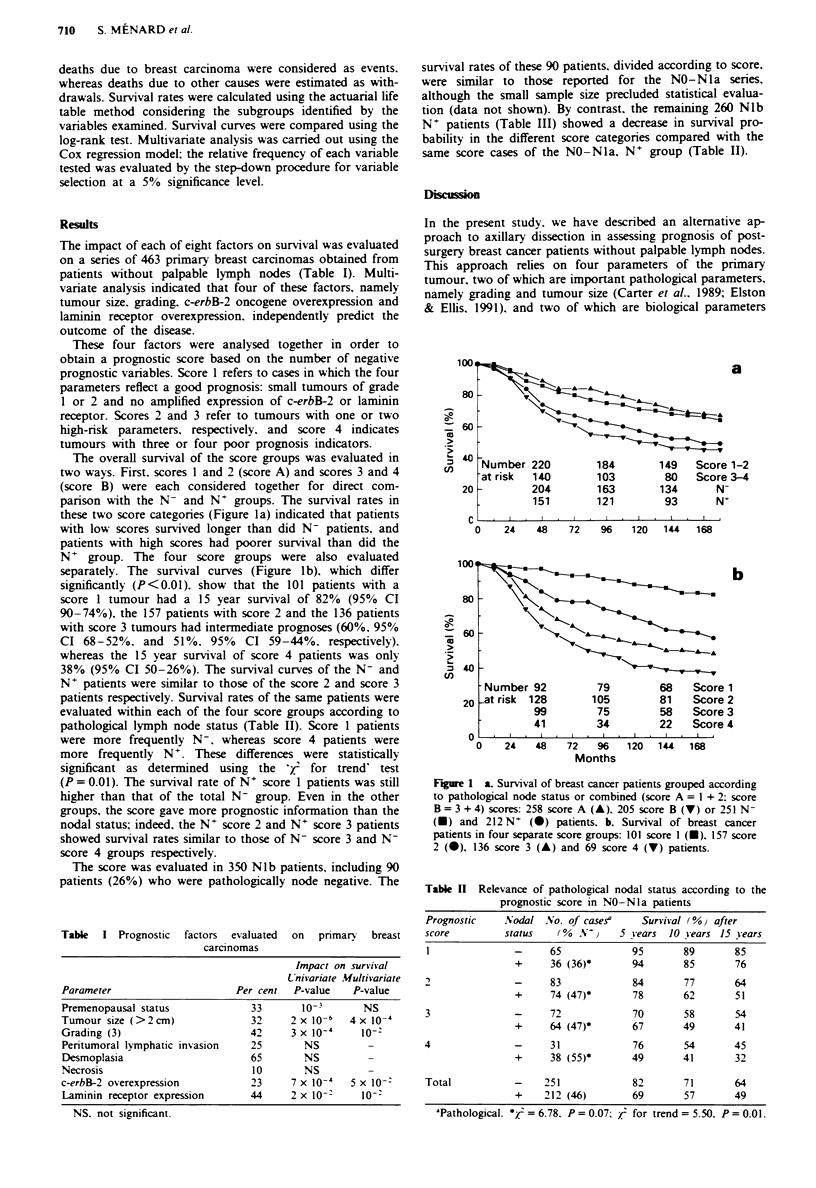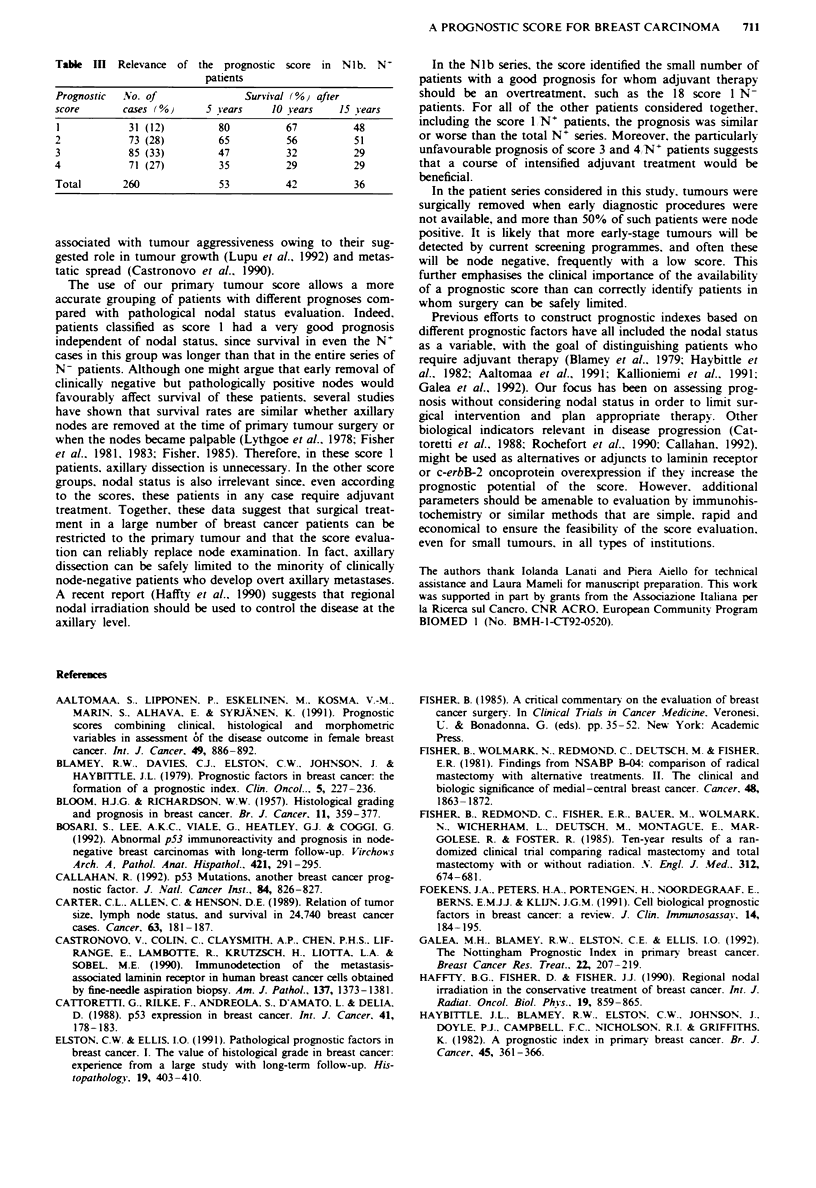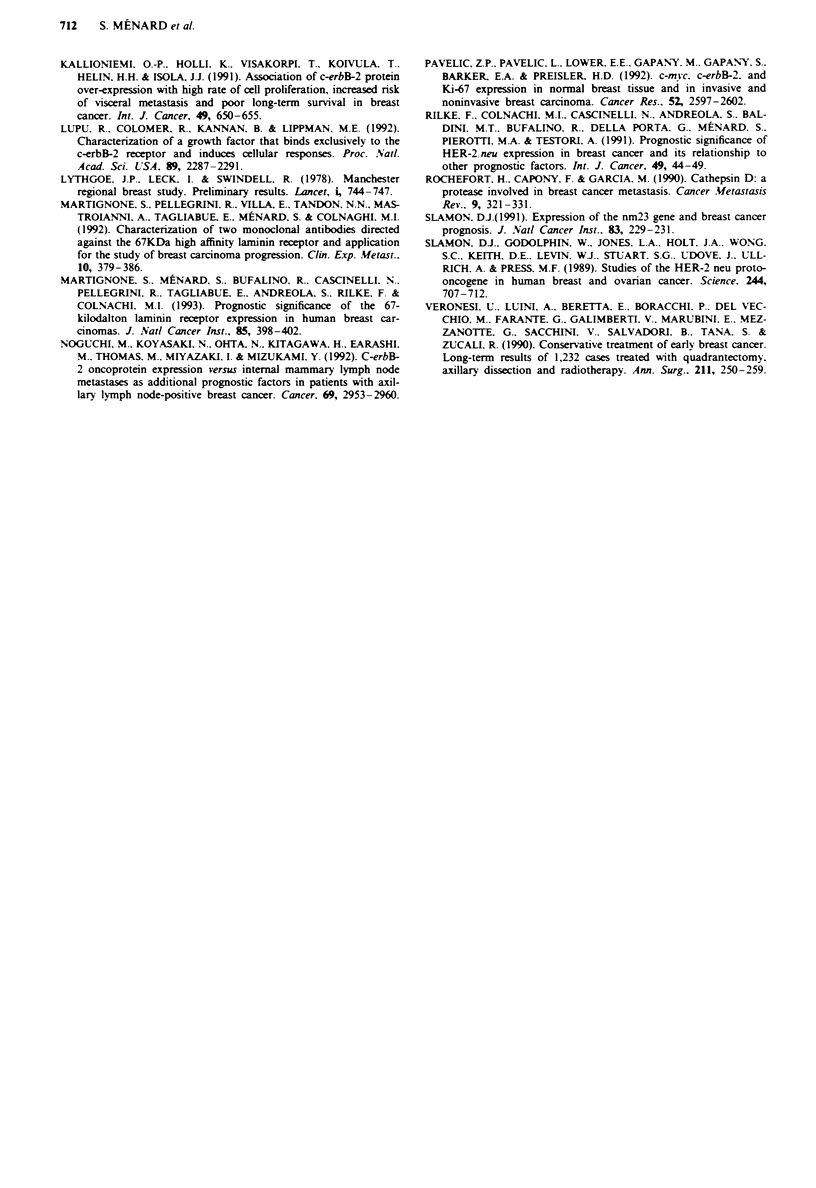# Prognosis based on primary breast carcinoma instead of pathological nodal status.

**DOI:** 10.1038/bjc.1994.379

**Published:** 1994-10

**Authors:** S. Ménard, R. Bufalino, F. Rilke, N. Cascinelli, U. Veronesi, M. I. Colnaghi

**Affiliations:** Department of Experimental Oncology E, Istituto Nazionale Tumori, Milan, Italy.

## Abstract

In breast cancer patients, prognostic information required to plan post-surgical therapy is obtained mainly through axillary dissection. This study was designed to establish a new prognostic score based solely on parameters of the primary tumour as an alternative to axillary surgery in assessing prognosis. Eight different prognostic factors, including menopausal status, tumour size, grading, lymphatic invasion, desmoplasia, necrosis, c-erbB-2 and laminin receptor expression, were evaluated retrospectively on a large series of primary breast carcinoma patients. From multivariate analysis, four independent parameters were selected and examined, alone and in combination, for their prognostic potential. These parameters were used to generate a prognostic score that was analysed retrospectively in 467 N0-N1a patients to determine its predictive value for survival. The score, which includes variables such as tumour size, grading, laminin receptor and c-erbB-2 overexpression, was established based on the number of negative prognostic factors: score 1 refers to cases in which all four parameters reflect a good prognosis, scores 2 and 3 refer to tumours in which, respectively, one or two of the four parameters reflect a poor prognosis, whereas score 4 refers to tumours with three or four poor prognosis factors. Analysis of the overall survival of the four score groups shows that patients with score 1 tumours (22% of the total) had the best prognosis with a 15 year survival of 82%, patients with score 2 and 3 had an intermediate prognosis, whereas score 4 patients had the poorest prognosis with a 15 year survival of only 38%. Moreover, survival in the N+ score 1 cases was found to be longer than that in the total N- patients. Our data suggest that the primary tumour score provides more reliable prognostic information than pathological nodal status, and that axillary dissection can be avoided in a large number of patients.


### Prognosis based on primary breast carcinoma instead of pathological nodal status

S. Ménard[1], R. Bufalino[2], F. Rilke[3], N. Cascinelli[2], U. Veronesi[4] & M.I. Colnaghi[1]

*Departments of [1]Experimental Oncology E, [2]Surgical Oncology B and [3]Anatomical Pathology and Cytology and [4]Scientific Director, Istituto Nazionale Tumori, Via Venezian 1, 20133 Milan, Italy.*

**Summary** In breast cancer patients, prognostic information required to plan post-surgical therapy is obtained mainly through axillary dissection. This study was designed to establish a new prognostic score based solely on parameters of the primary tumour as an alternative to axillary surgery in assessing prognosis. Eight different prognostic factors, including menopausal status, tumour size, grading, lymphatic invasion, desmoplasia, necrosis, c-*erb*B-2 and laminin receptor expression, were evaluated retrospectively on a large series of primary breast carcinoma patients. From multivariate analysis, four independent parameters were selected and examined, alone and in combination, for their prognostic potential. These parameters were used to generate a prognostic score that was analysed retrospectively in 467 N0-N1a patients to determine its predictive value for survival. The score, which includes variables such as tumour size, grading, laminin receptor and c-*erb*B-2 overexpression, was established based on the number of negative prognostic factors: score 1 refers to cases in which all four parameters reflect a good prognosis, scores 2 and 3 refer to tumours in which, respectively, one or two of the four parameters reflect a poor prognosis, whereas score 4 refers to tumours with three or four poor prognosis factors. Analysis of the overall survival of the four score groups shows that patients with score 1 tumours (22% of the total) had the best prognosis with a 15 year survival of 82%, patients with score 2 and 3 had an intermediate prognosis, whereas score 4 patients had the poorest prognosis with a 15 year survival of only 38%. Moreover, survival in the $N^+$ score 1 cases was found to be longer than that in the total $N^-$ patients. Our data suggest that the primary tumour score provides more reliable prognostic information than pathological nodal status, and that axillary dissection can be avoided in a large number of patients.

Post-surgical treatment of breast cancer patients is determined largely on the basis of pathological nodal status, which is considered to be the most important prognostic indicator of this disease. In the past, most tumours were diagnosed at an advanced stage, with frequent nodal involvement, so that axillary dissection was performed for both prognostic and therapeutic purposes. However, with current screening programmes for early breast carcinoma detection, the frequency of patients presenting with nodal involvement is considerably decreased. As a result, surgical intervention on the axillae is performed primarily to obtain prognostic information rather than to control regional disease (Fisher *et al.*, 1985). In the absence of alternative prognostic factors, nodal status remains critical in identifying patients who require adjuvant systemic therapy. Thus, despite that recent trend toward less invasive surgical intervention for the primary tumour (Veronesi *et al.*, 1990), the advantages of breast conservative surgery are limited by axillary node dissection, which is still routinely done.

The concept that tumour aggressiveness can be evaluated not only by analysing parameters that measure a metastatic event, such as nodal spread, but also by analysing intrinsic biological factors displayed by the primary tumour, has been widely investigated (Foekens *et al.*, 1991; Slamon, 1991; Bosari *et al.*, 1992; Noguchi *et al.*, 1992; Pavelic *et al.*, 1992). However, so far no single prognostic factor, such as oncogenes, suppressor genes, enzymes or adhesion receptors, has been found to be as potent a predictor as nodal status (Slamon *et al.*, 1989; Rilke *et al.*, 1991; Martignone *et al.*, 1993). Unfortunately, nodal status in some cases fail to correctly predict the prognosis; in fact, 30% of $N^-$ patients relapse and 30% of $N^+$ patients have a long survival (Galea *et al.*, 1992).

In the present study, we describe a prognostic score based only on parameters of the primary tumour that may avoid the need for axillary dissection in clinically node-negative patients. This score, evaluated retrospectively on 463 primary breast carcinomas from patients without palpable nodes, appears to provide more accurate prognostic information than does nodal status. In addition, the score was evaluated in 350 N1b patients in association with nodal status and it was found to identify those patients in whom nodal status failed to predict the correct prognosis.

Correspondence: S. Ménard.

Received 18 February 1994; and in revised form 13 May 1994.

### Patients and methods

### *Patients*

The study included 813 patients surgically treated at this institute from 1968 to 1969 for infiltrating duct or lobular breast carcinoma. Surgical treatment consisted of radical or modified radical mastectomy and axillary dissection. Only histologically node-positive patients received post-surgical radiotherapy on supraclavicular and internal mammary lymph nodes. No patient had adjuvant systemic therapy. Perimenopausal patients were classified as premenopausal.

### *Histopathology*

Tumour size and nodal infiltration were obtained from histopathological reports. The grading procedure was performed according to Bloom and Richardson (1957) and grades I and II were considered together for the score evaluation. Peritumoral lymphatic invasion, desmoplasia and necrosis were evaluated as previously described (Rilke *et al.*, 1991).

### *Immunohistochemistry*

Paraffin sections were stained as previously described (Rilke *et al.*, 1991) using the avidin-biotin-peroxidase method and antibodies directed against the c-*erb*B-2 oncoprotein (Slamon *et al.*, 1989) or the laminin receptor (Martignone *et al.*, 1992). Sections were considered positive when more than 10% of the tumour cells were labelled.

### *Statistical analysis*

Overall survival of patients from the date of surgical treatment was considered as the end point of this study. Only deaths due to breast carcinoma were considered as events, whereas deaths due to other causes were estimated as withdrawals. Survival rates were calculated using the actuarial life table method considering the subgroups identified by the variables examined. Survival curves were compared using the log-rank test. Multivariate analysis was carried out using the Cox regression model; the relative frequency of each variable tested was evaluated by the step-down procedure for variable selection at a 5% significance level.

### Results

The impact of each of eight factors on survival was evaluated on a series of 463 primary breast carcinomas obtained from patients without palpable lymph nodes (Table I). Multivariate analysis indicated that four of these factors, namely tumour size, grading, c-*erb*B-2 oncogene overexpression and laminin receptor overexpression, independently predict the outcome of the disease.

These four factors were analysed together in order to obtain a prognostic score based on the number of negative prognostic variables. Score 1 refers to cases in which the four parameters reflect a good prognosis: small tumours of grade 1 or 2 and no amplified expression of c-*erb*B-2 or laminin receptor. Scores 2 and 3 refer to tumours with one or two high-risk parameters, respectively, and score 4 indicates tumours with three or four poor prognosis indicators.

The overall survival of the score groups was evaluated in two ways. First, scores 1 and 2 (score A) and scores 3 and 4 (score B) were each considered together for direct comparison with the $N^-$ and $N^+$ groups. The survival rates in these two score categories (Figure 1a) indicated that patients with low scores survived longer than did $N^-$ patients, and patients with high scores had poorer survival than did the $N^+$ group. The four score groups were also evaluated separately. The survival curves (Figure 1b), which differ significantly ($P<0.01$), show that the 101 patients with a score 1 tumour had a 15 year survival of 82% (95% CI 90–74%), the 157 patients with score 2 and the 136 patients with score 3 tumours had intermediate prognoses (60%, 95% CI 68–52%, and 51%, 95% CI 59–44%, respectively), whereas the 15 year survival of score 4 patients was only 38% (95% CI 50–26%). The survival curves of the $N^-$ and $N^+$ patients were similar to those of the score 2 and score 3 patients respectively. Survival rates of the same patients were evaluated within each of the four score groups according to pathological lymph node status (Table II). Score 1 patients were more frequently $N^-$, whereas score 4 patients were more frequently $N^+$. These differences were statistically significant as determined using the '$\chi^2$ for trend' test ($P=0.01$). The survival rate of $N^+$ score 1 patients was still higher than that of the total $N^-$ group. Even in the other groups, the score gave more prognostic information than the nodal status; indeed, the $N^+$ score 2 and $N^+$ score 3 patients showed survival rates similar to those of $N^-$ score 3 and $N^-$ score 4 groups respectively.

The score was evaluated in 350 N1b patients, including 90 patients (26%) who were pathologically node negative. The survival rates of these 90 patients, divided according to score, were similar to those reported for the N0–N1a series, although the small sample size precluded statistical evaluation (data not shown). By contrast, the remaining 260 N1b $N^+$ patients (Table III) showed a decrease in survival probability in the different score categories compared with the same score cases of the N0–N1a, $N^+$ group (Table II).

**Table I** Prognostic factors evaluated on primary breast carcinomas

| Parameter | Per cent | Impact on survival: Univariate *P*-value | Impact on survival: Multivariate *P*-value |
|---|---|---|---|
| Premenopausal status | 33 | $10^{-3}$ | NS |
| Tumour size (>2 cm) | 32 | $2\times10^{-6}$ | $4\times10^{-4}$ |
| Grading (3) | 42 | $3\times10^{-4}$ | $10^{-2}$ |
| Peritumoral lymphatic invasion | 25 | NS | – |
| Desmoplasia | 65 | NS | – |
| Necrosis | 10 | NS | – |
| c-*erb*B-2 overexpression | 23 | $7\times10^{-4}$ | $5\times10^{-2}$ |
| Laminin receptor expression | 44 | $2\times10^{-2}$ | $10^{-2}$ |

NS, not significant.

### Discussion

In the present study, we have described an alternative approach to axillary dissection in assessing prognosis of post-surgery breast cancer patients without palpable lymph nodes. This approach relies on four parameters of the primary tumour, two of which are important pathological parameters, namely grading and tumour size (Carter *et al.*, 1989; Elston & Ellis, 1991), and two of which are biological parameters

**Figure 1** **a**, Survival of breast cancer patients grouped according to pathological node status or combined (score A = 1 + 2; score B = 3 + 4) scores: 258 score A (▲), 205 score B (▼) or 251 $N^-$ (■) and 212 $N^+$ (●) patients. **b**, Survival of breast cancer patients in four separate score groups: 101 score 1 (■), 157 score 2 (●), 136 score 3 (▲) and 69 score 4 (▼) patients.

**Table II** Relevance of pathological nodal status according to the prognostic score in N0–N1a patients

| Prognostic score | Nodal status | No. of cases[a] (% $N^+$) | Survival (%) after 5 years | 10 years | 15 years |
|---|---|---|---|---|---|
| 1 | – | 65 | 95 | 89 | 85 |
| | + | 36 (36)* | 94 | 85 | 76 |
| 2 | – | 83 | 84 | 77 | 64 |
| | + | 74 (47)* | 78 | 62 | 51 |
| 3 | – | 72 | 70 | 58 | 54 |
| | + | 64 (47)* | 67 | 49 | 41 |
| 4 | – | 31 | 76 | 54 | 45 |
| | + | 38 (55)* | 49 | 41 | 32 |
| Total | – | 251 | 82 | 71 | 64 |
| | + | 212 (46) | 69 | 57 | 49 |

[a]Pathological. *$\chi^2=6.78$, $P=0.07$; $\chi^2$ for trend = 5.50, $P=0.01$.

**Table III** Relevance of the prognostic score in N1b. N$^+$ patients

| *Prognostic score* | *No. of cases (%)* | *Survival (%) after* | | |
|---|---|---|---|---|
| | | *5 years* | *10 years* | *15 years* |
| 1 | 31 (12) | 80 | 67 | 48 |
| 2 | 73 (28) | 65 | 56 | 51 |
| 3 | 85 (33) | 47 | 32 | 29 |
| 4 | 71 (27) | 35 | 29 | 29 |
| Total | 260 | 53 | 42 | 36 |

associated with tumour aggressiveness owing to their suggested role in tumour growth (Lupu *et al.*, 1992) and metastatic spread (Castronovo *et al.*, 1990).

The use of our primary tumour score allows a more accurate grouping of patients with different prognoses compared with pathological nodal status evaluation. Indeed, patients classified as score 1 had a very good prognosis independent of nodal status, since survival in even the N$^+$ cases in this group was longer than that in the entire series of N$^-$ patients. Although one might argue that early removal of clinically negative but pathologically positive nodes would favourably affect survival of these patients, several studies have shown that survival rates are similar whether axillary nodes are removed at the time of primary tumour surgery or when the nodes became palpable (Lythgoe *et al.*, 1978; Fisher *et al.*, 1981, 1983; Fisher, 1985). Therefore, in these score 1 patients, axillary dissection is unnecessary. In the other score groups, nodal status is also irrelevant since, even according to the scores, these patients in any case require adjuvant treatment. Together, these data suggest that surgical treatment in a large number of breast cancer patients can be restricted to the primary tumour and that the score evaluation can reliably replace node examination. In fact, axillary dissection can be safely limited to the minority of clinically node-negative patients who develop overt axillary metastases. A recent report (Haffty *et al.*, 1990) suggests that regional nodal irradiation should be used to control the disease at the axillary level.

In the N1b series, the score identified the small number of patients with a good prognosis for whom adjuvant therapy should be an overtreatment, such as the 18 score 1 N$^-$ patients. For all of the other patients considered together, including the score 1/N$^+$ patients, the prognosis was similar or worse than the total N$^+$ series. Moreover, the particularly unfavourable prognosis of score 3 and 4/N$^+$ patients suggests that a course of intensified adjuvant treatment would be beneficial.

In the patient series considered in this study, tumours were surgically removed when early diagnostic procedures were not available, and more than 50% of such patients were node positive. It is likely that more early-stage tumours will be detected by current screening programmes, and often these will be node negative, frequently with a low score. This further emphasises the clinical importance of the availability of a prognostic score than can correctly identify patients in whom surgery can be safely limited.

Previous efforts to construct prognostic indexes based on different prognostic factors have all included the nodal status as a variable, with the goal of distinguishing patients who require adjuvant therapy (Blamey *et al.*, 1979; Haybittle *et al.*, 1982; Aaltomaa *et al.*, 1991; Kallioniemi *et al.*, 1991; Galea *et al.*, 1992). Our focus has been on assessing prognosis without considering nodal status in order to limit surgical intervention and plan appropriate therapy. Other biological indicators relevant in disease progression (Cattoretti *et al.*, 1988; Rochefort *et al.*, 1990; Callahan, 1992), might be used as alternatives or adjuncts to laminin receptor or c-*erb*B-2 oncoprotein overexpression if they increase the prognostic potential of the score. However, additional parameters should be amenable to evaluation by immunohistochemistry or similar methods that are simple, rapid and economical to ensure the feasibility of the score evaluation, even for small tumours, in all types of institutions.

The authors thank Iolanda Lanati and Piera Aiello for technical assistance and Laura Mameli for manuscript preparation. This work was supported in part by grants from the Associazione Italiana per la Ricerca sul Cancro, CNR ACRO, European Community Program BIOMED 1 (No. BMH-1-CT92-0520).

### References

AALTOMAA, S., LIPPONEN, P., ESKELINEN, M., KOSMA, V.-M., MARIN, S., ALHAVA, E. & SYRJÄNEN, K. (1991). Prognostic scores combining clinical, histological and morphometric variables in assessment of the disease outcome in female breast cancer. *Int. J. Cancer*, **49**, 886-892.

BLAMEY, R.W., DAVIES, C.J., ELSTON, C.W., JOHNSON, J. & HAYBITTLE, J.L. (1979). Prognostic factors in breast cancer: the formation of a prognostic index. *Clin. Oncol.*, **5**, 227-236.

BLOOM, H.J.G. & RICHARDSON, W.W. (1957). Histological grading and prognosis in breast cancer. *Br. J. Cancer*, **11**, 359-377.

BOSARI, S., LEE, A.K.C., VIALE, G., HEATLEY, G.J. & COGGI, G. (1992). Abnormal *p53* immunoreactivity and prognosis in node-negative breast carcinomas with long-term follow-up. *Virchows Arch. A, Pathol. Anat. Hispathol.*, **421**, 291-295.

CALLAHAN, R. (1992). p53 Mutations, another breast cancer prognostic factor. *J. Natl. Cancer Inst.*, **84**, 826-827.

CARTER, C.L., ALLEN, C. & HENSON, D.E. (1989). Relation of tumor size, lymph node status, and survival in 24,740 breast cancer cases. *Cancer*, **63**, 181-187.

CASTRONOVO, V., COLIN, C., CLAYSMITH, A.P., CHEN, P.H.S., LIFRANGE, E., LAMBOTTE, R., KRUTZSCH, H., LIOTTA, L.A. & SOBEL, M.E. (1990). Immunodetection of the metastasis-associated laminin receptor in human breast cancer cells obtained by fine-needle aspiration biopsy. *Am. J. Pathol.*, **137**, 1373-1381.

CATTORETTI, G., RILKE, F., ANDREOLA, S., D'AMATO, L. & DELIA, D. (1988). p53 expression in breast cancer. *Int. J. Cancer*, **41**, 178-183.

ELSTON, C.W. & ELLIS, I.O. (1991). Pathological prognostic factors in breast cancer. I. The value of histological grade in breast cancer: experience from a large study with long-term follow-up. *Histopathology*, **19**, 403-410.

FISHER, B. (1985). A critical commentary on the evaluation of breast cancer surgery. In *Clinical Trials in Cancer Medicine*. Veronesi, U. & Bonadonna, G. (eds). pp. 35-52. New York: Academic Press.

FISHER, B., WOLMARK, N., REDMOND, C., DEUTSCH, M. & FISHER, E.R. (1981). Findings from NSABP B-04: comparison of radical mastectomy with alternative treatments. II. The clinical and biologic significance of medial-central breast cancer. *Cancer*, **48**, 1863-1872.

FISHER, B., REDMOND, C., FISHER, E.R., BAUER, M., WOLMARK, N., WICHERHAM, L., DEUTSCH, M., MONTAGUE, E., MARGOLESE, R. & FOSTER, R. (1985). Ten-year results of a randomized clinical trial comparing radical mastectomy and total mastectomy with or without radiation. *N. Engl. J. Med.*, **312**, 674-681.

FOEKENS, J.A., PETERS, H.A., PORTENGEN, H., NOORDEGRAAF, E., BERNS, E.M.J.J. & KLIJN, J.G.M. (1991). Cell biological prognostic factors in breast cancer: a review. *J. Clin. Immunosassay*, **14**, 184-195.

GALEA, M.H., BLAMEY, R.W., ELSTON, C.E. & ELLIS, I.O. (1992). The Nottingham Prognostic Index in primary breast cancer. *Breast Cancer Res. Treat.*, **22**, 207-219.

HAFFTY, B.G., FISHER, D. & FISHER, J.J. (1990). Regional nodal irradiation in the conservative treatment of breast cancer. *Int. J. Radiat. Oncol. Biol. Phys.*, **19**, 859-865.

HAYBITTLE, J.L., BLAMEY, R.W., ELSTON, C.W., JOHNSON, J., DOYLE, P.J., CAMPBELL, F.C., NICHOLSON, R.I. & GRIFFITHS, K. (1982). A prognostic index in primary breast cancer. *Br. J. Cancer*, **45**, 361-366.

KALLIONIEMI, O.-P., HOLLI, K., VISAKORPI, T., KOIVULA, T., HELIN, H.H. & ISOLA, J.J. (1991). Association of c-*erb*B-2 protein over-expression with high rate of cell proliferation, increased risk of visceral metastasis and poor long-term survival in breast cancer. *Int. J. Cancer*, **49**, 650-655.

LUPU, R., COLOMER, R., KANNAN, B. & LIPPMAN, M.E. (1992). Characterization of a growth factor that binds exclusively to the c-erbB-2 receptor and induces cellular responses. *Proc. Natl. Acad. Sci. USA*, **89**, 2287-2291.

LYTHGOE, J.P., LECK, I. & SWINDELL, R. (1978). Manchester regional breast study. Preliminary results. *Lancet*, **i**, 744-747.

MARTIGNONE, S., PELLEGRINI, R., VILLA, E., TANDON, N.N., MASTROIANNI, A., TAGLIABUE, E., MÉNARD, S. & COLNAGHI, M.I. (1992). Characterization of two monoclonal antibodies directed against the 67KDa high affinity laminin receptor and application for the study of breast carcinoma progression. *Clin. Exp. Metast.*, **10**, 379-386.

MARTIGNONE, S., MÉNARD, S., BUFALINO, R., CASCINELLI, N., PELLEGRINI, R., TAGLIABUE, E., ANDREOLA, S., RILKE, F. & COLNACHI, M.I. (1993). Prognostic significance of the 67-kilodalton laminin receptor expression in human breast carcinomas. *J. Natl Cancer Inst.*, **85**, 398-402.

NOGUCHI, M., KOYASAKI, N., OHTA, N., KITAGAWA, H., EARASHI, M., THOMAS, M., MIYAZAKI, I. & MIZUKAMI, Y. (1992). C-*erb*B-2 oncoprotein expression *versus* internal mammary lymph node metastases as additional prognostic factors in patients with axillary lymph node-positive breast cancer. *Cancer*, **69**, 2953-2960.

PAVELIC, Z.P., PAVELIC, L., LOWER, E.E., GAPANY, M., GAPANY, S., BARKER, E.A. & PREISLER, H.D. (1992). c-*myc*, c-*erb*B-2, and Ki-67 expression in normal breast tissue and in invasive and noninvasive breast carcinoma. *Cancer Res.*, **52**, 2597-2602.

RILKE, F., COLNACHI, M.I., CASCINELLI, N., ANDREOLA, S., BALDINI, M.T., BUFALINO, R., DELLA PORTA, G., MÉNARD, S., PIEROTTI, M.A. & TESTORI, A. (1991). Prognostic significance of HER-2/*neu* expression in breast cancer and its relationship to other prognostic factors. *Int. J. Cancer*, **49**, 44-49.

ROCHEFORT, H., CAPONY, F. & GARCIA, M. (1990). Cathepsin D: a protease involved in breast cancer metastasis. *Cancer Metastasis Rev.*, **9**, 321-331.

SLAMON, D.J.(1991). Expression of the nm23 gene and breast cancer prognosis. *J. Natl Cancer Inst.*, **83**, 229-231.

SLAMON, D.J., GODOLPHIN, W., JONES, L.A., HOLT, J.A., WONG, S.C., KEITH, D.E., LEVIN, W.J., STUART, S.G., UDOVE, J., ULLRICH, A. & PRESS, M.F. (1989). Studies of the HER-2 neu protooncogene in human breast and ovarian cancer. *Science*, **244**, 707-712.

VERONESI, U., LUINI, A., BERETTA, E., BORACCHI, P., DEL VECCHIO, M., FARANTE, G., GALIMBERTI, V., MARUBINI, E., MEZZANOTTE, G., SACCHINI, V., SALVADORI, B., TANA, S. & ZUCALI, R. (1990). Conservative treatment of early breast cancer. Long-term results of 1,232 cases treated with quadrantectomy, axillary dissection and radiotherapy. *Ann. Surg.*, **211**, 250-259.